# Supplementary material for: Effects of Auxin (Indole-3-butyric Acid) on Adventitious Root Formation in Peach-Based Prunus Rootstocks
Source: Plants (Basel). 2022 Mar 29;11(7):913. doi: 10.3390/plants11070913 (PMC9002465; doi:10.3390/plants11070913)
Supplement: Supplementary file 1 [file plants-11-00913-s001.zip › plants-1652499-supplementary/PRUNUS_FigureS1.pdf]

Figure S1

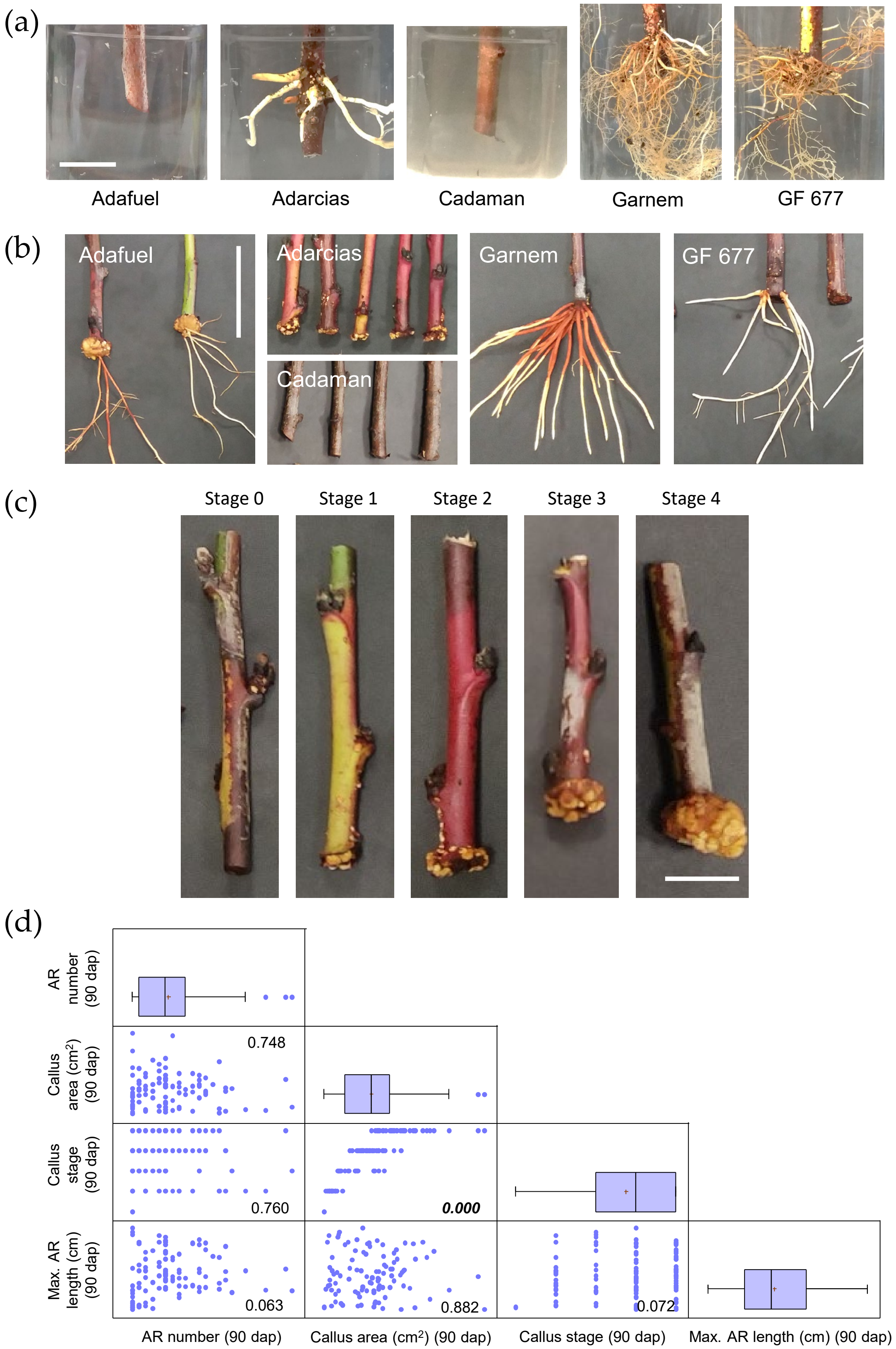

Figure S1. Regenerative response of studied *Prunus* rootstocks and correlation among different regeneration parameters. (**a**) Representative images of five *Prunus* rootstocks at 82 days after growing in soil conditions. Scale bar: 2.5 cm. (**b**) Representative images of the basal part of studied *Prunus* rootstocks at 90 dap. Scale bar: 5 cm. (**c**) Different callus stages were stablished in order to define the regeneration capacity at the basal part of the cuttings. Scale bar: 15 mm. (**d**) Multiple correlation analysis was performed among regeneration parameters measured at 90 dap after hydroponic culture, with only a statistically significant correlation (p-value < 0.05) between callus area and callus stage parameters.
