# Supplementary material for: Effects of Auxin (Indole-3-butyric Acid) on Adventitious Root Formation in Peach-Based Prunus Rootstocks
Source: Plants (Basel). 2022 Mar 29;11(7):913. doi: 10.3390/plants11070913 (PMC9002465; doi:10.3390/plants11070913)
Supplement: Supplementary file 1 [file plants-11-00913-s001.zip › plants-1652499-supplementary/PRUNUS_FigureS2.pdf]

Figure S2

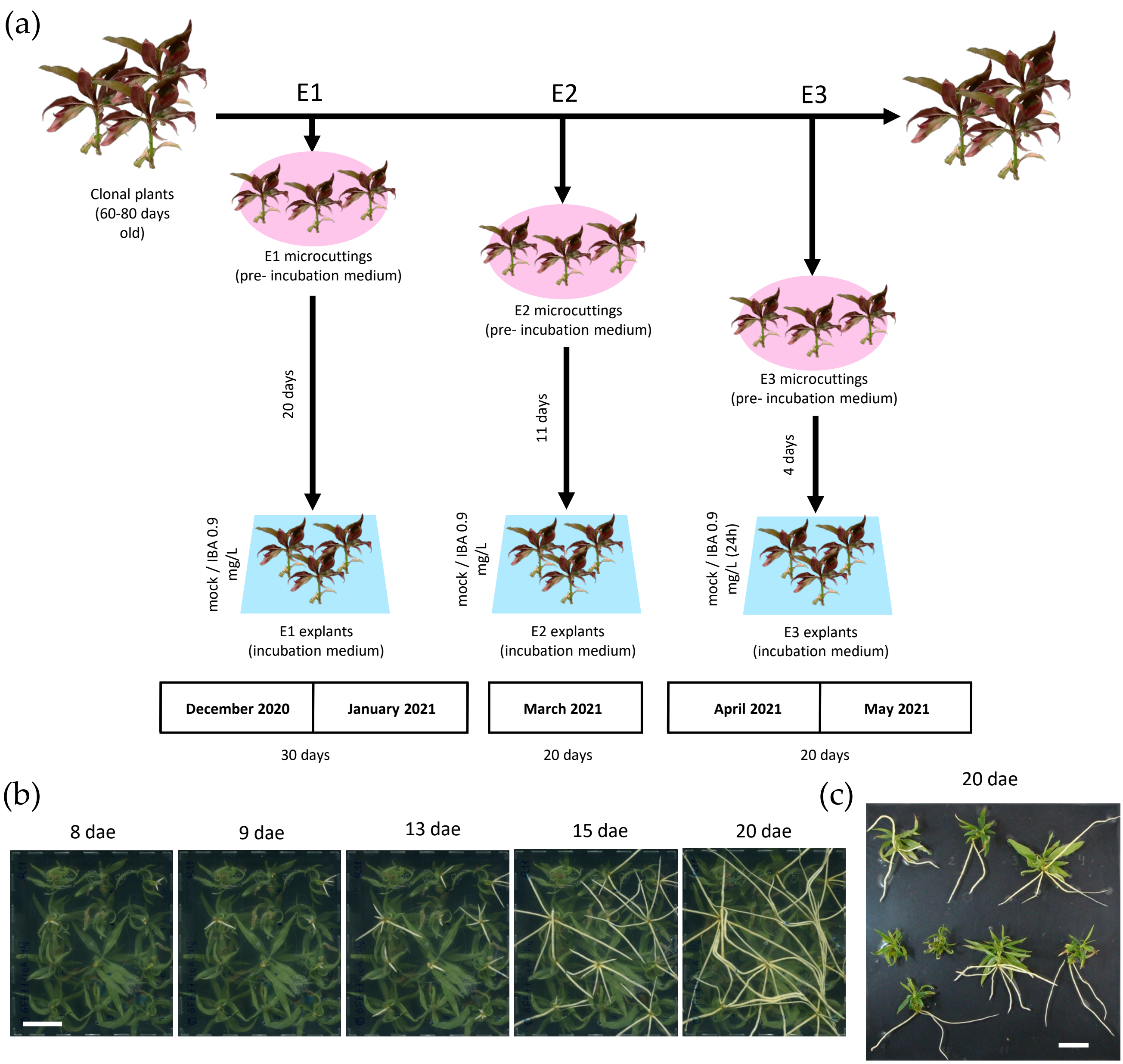

Figure S2. Adventitious rooting evaluation in *Prunus* at *in vitro* culture. (**a**) Experimental design stablished for adventitious root characterization in Garnem and GF 677 microcuttings at *in vitro* growing conditions by three different experiments (E1, E2 and E3). (**b**) Representative images of the time-series scans at different time points at *in vitro* experiments and (**c**) final images of regenerated microcuttings. Scale bars 25 mm.
