# Supplementary material for: Effects of Auxin (Indole-3-butyric Acid) on Adventitious Root Formation in Peach-Based Prunus Rootstocks
Source: Plants (Basel). 2022 Mar 29;11(7):913. doi: 10.3390/plants11070913 (PMC9002465; doi:10.3390/plants11070913)
Supplement: Supplementary file 1 [file plants-11-00913-s001.zip › plants-1652499-supplementary/PRUNUS_FigureS3.pdf]

Figure S3

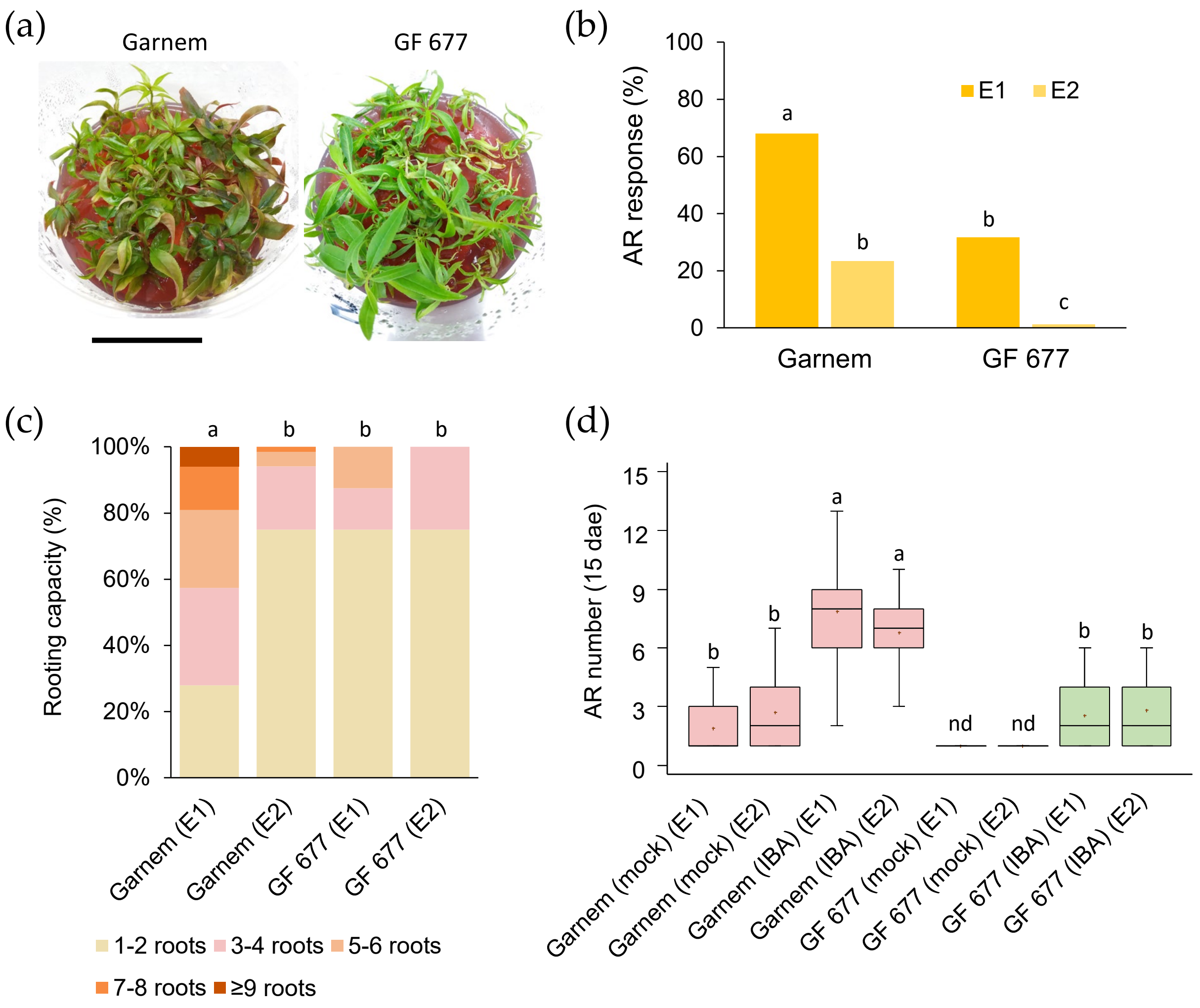

Figure S3. AR formation in Garnem and GF 677 microcuttings. (**a**) Garnem (left) and GF 677 (right) microcuttings in the pre-incubation medium before excision. Scale bar: 5 cm. (**b**) AR response after 20 days in the pre-incubation for microcuttings used in the first experimental replicate (E1) and after 11 days for microcuttings used in the second experimental replicate (E2). Sample number in AR response percentages ranges from 97 to 309. Letters indicate significant differences (p-value < 0.05) among genotypes and replicates. (**c**) Rooting capacity of microcuttings of both genotypes used for E1 and E2 before excision in the pre-incubation medium. Sample number in rooting capacity graph ranges from 4 to 68. Letters indicate significant differences (p-value < 0.01) among genotypes for average AR number. (**d**) Adventitious root number in Garnem and GF 677 at 15 days after excision (dae) for E1 and E2. Sample number in AR number graph ranges from 11 to 34. Letters indicate significant differences (p-value < 0.01) among genotypes and treatments, nd = without data.
