# Supplementary material for: Effects of Auxin (Indole-3-butyric Acid) on Adventitious Root Formation in Peach-Based Prunus Rootstocks
Source: Plants (Basel). 2022 Mar 29;11(7):913. doi: 10.3390/plants11070913 (PMC9002465; doi:10.3390/plants11070913)
Supplement: Supplementary file 1 [file plants-11-00913-s001.zip › plants-1652499-supplementary/PRUNUS_FigureS4.pdf]

Figure S4

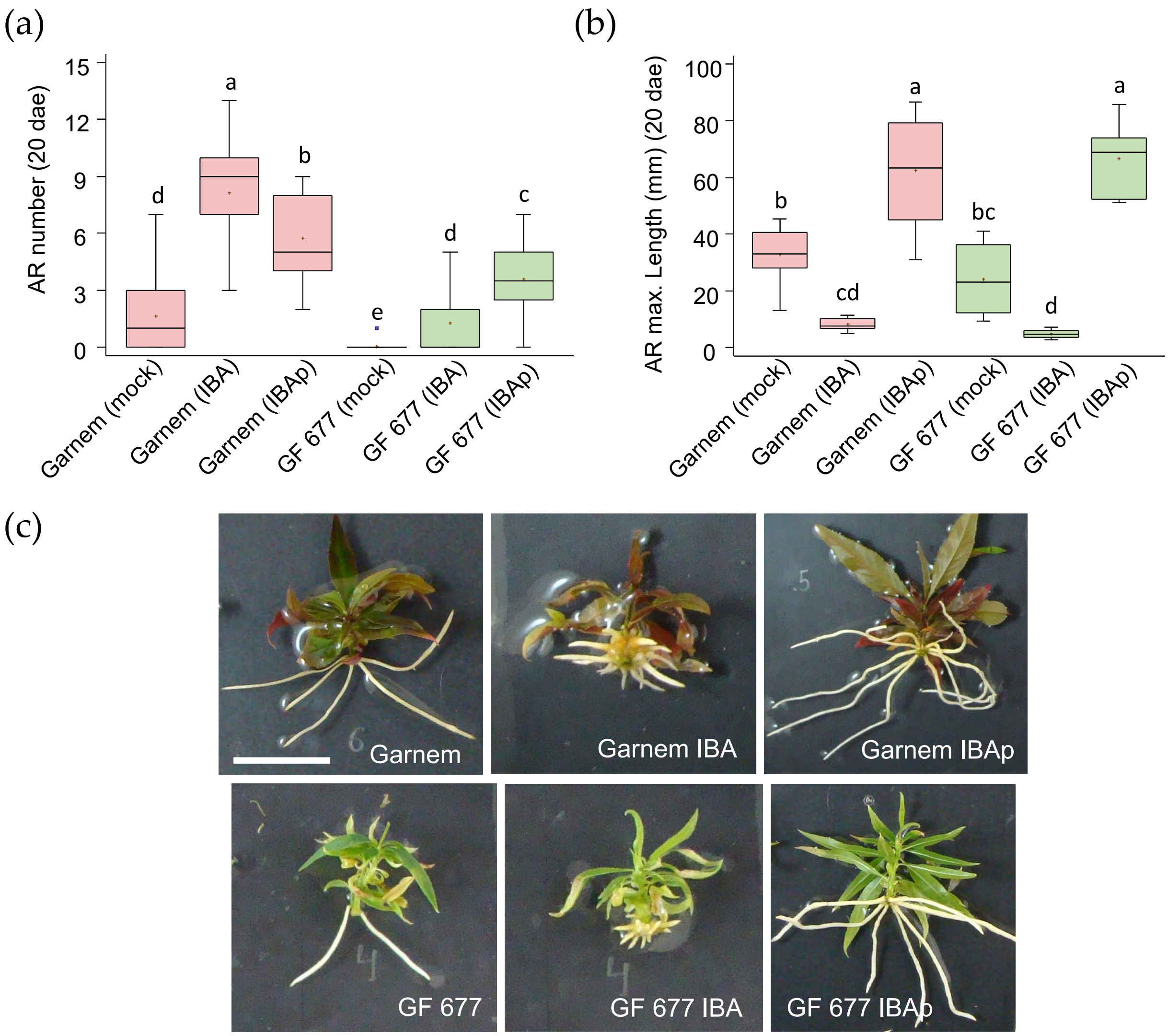

Figure S4. AR root formation in *Prunus* microcuttings with different treatments. (**a**) AR number in Garnem and GF 677 microcuttings with mock, IBA or IBA-pulse (IBAp) treatments at 20 days after excision (dae). (B) AR maximum length in Garnem and GF 677 microcuttings with mock, IBA or IBAp treatments at 20 dae. Letters indicate significant differences (p-value < 0.01) among genotypes. Sample number ranges from 8 to 75. (C) Representative images of Garnem and GF 677 genotypes with different treatments at the end of the experiment. Scale bar: 2.5 cm.
