# Supplementary material for: Effects of Auxin (Indole-3-butyric Acid) on Adventitious Root Formation in Peach-Based Prunus Rootstocks
Source: Plants (Basel). 2022 Mar 29;11(7):913. doi: 10.3390/plants11070913 (PMC9002465; doi:10.3390/plants11070913)
Supplement: Supplementary file 1 [file plants-11-00913-s001.zip › plants-1652499-supplementary/PRUNUS_FigureS5.pdf]

Figure S5

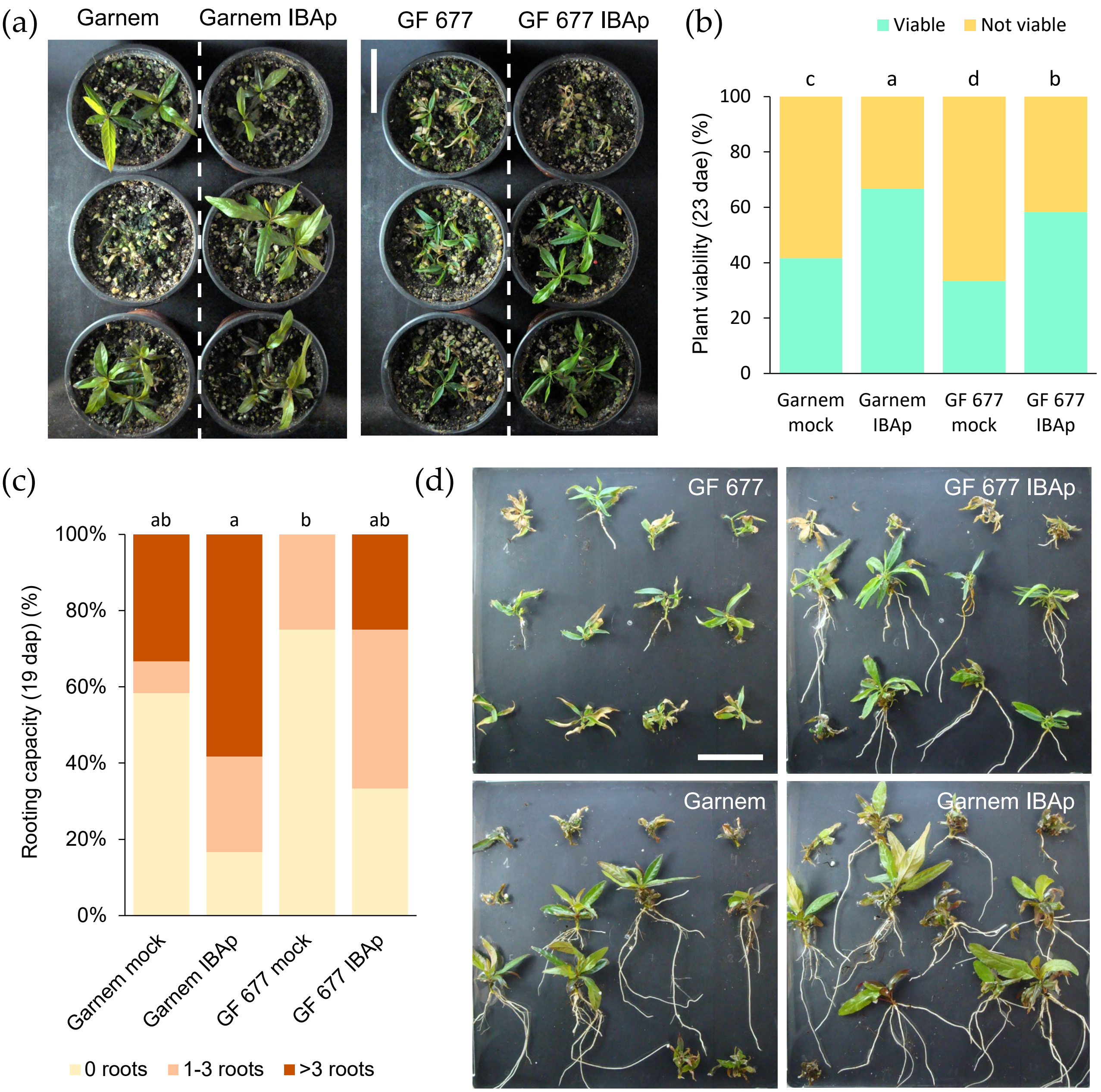

Figure S5. *Ex vitro* acclimation of *Prunus* microcuttings after IBAp treatment. (**a**) Plant growing after four days at *in vitro* conditions and 19 days at soil conditions. Scale bar: 5 cm. (**b**) Plant viability percentage in each condition. (**c**) Rooting capacity of each genotype and condition after 19 dap / 23 dae. Letters indicate significant differences (p-value < 0.01) for the average AR number between the genotypes studied. Sample number is 12. (**d**) Representative images of the four studied conditions. Scale bar: 5 cm.
