# Supplementary material for: Effects of Auxin (Indole-3-butyric Acid) on Adventitious Root Formation in Peach-Based Prunus Rootstocks
Source: Plants (Basel). 2022 Mar 29;11(7):913. doi: 10.3390/plants11070913 (PMC9002465; doi:10.3390/plants11070913)
Supplement: Supplementary file 1 [file plants-11-00913-s001.zip › plants-1652499-supplementary/PRUNUS_FigureS6.pdf]

# Figure S6

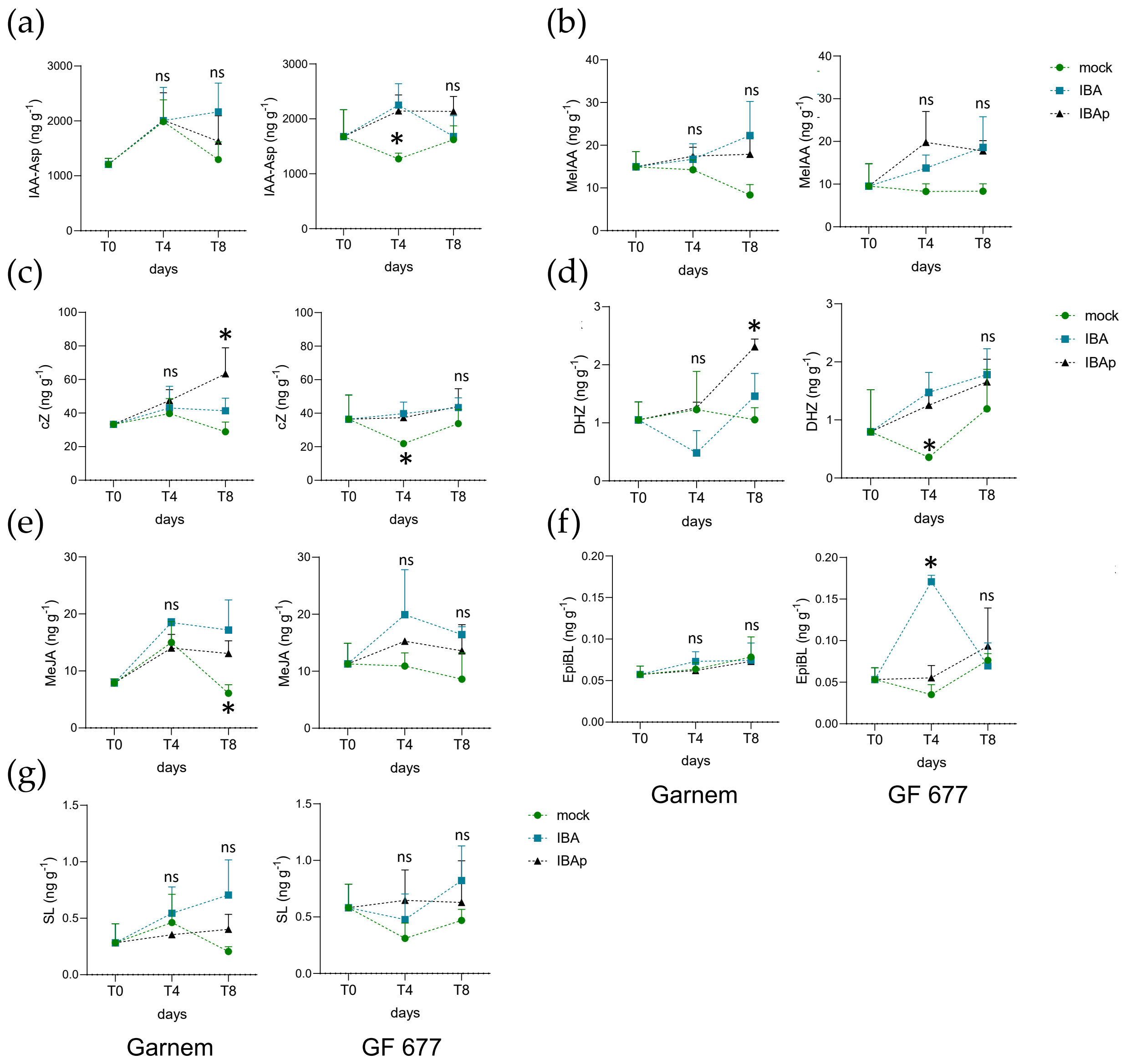


**Figure S6.** Hormonal profiling of Garnem and GF 677 microcuttings during IBA-induced rooting (II). Endogenous levels of (**a**) indole-3-acetyl-aspartate (IAA-Asp), (**b**) methyl indole-3-acetic acid (MeIAA), (**c**) cis-zeatin (cZ), (**d**) dihidrozeatin (DHZ), (**e**) methyl jasmonate (MeJA), (**f**) epi-brassinolide (EpiBL) and (**g**) solanacol (SL) measured in the basal region of Garnem (left panels) and GF 677 (right panels) microcuttings at 0, 4 and 8 dae. Asterisks indicate statistically significant differences (p-value < 0.01) among treatments at the same time point, ns= no statistically significant differences (p-value < 0.01).
