# Supplementary material for: Effects of Auxin (Indole-3-butyric Acid) on Adventitious Root Formation in Peach-Based Prunus Rootstocks
Source: Plants (Basel). 2022 Mar 29;11(7):913. doi: 10.3390/plants11070913 (PMC9002465; doi:10.3390/plants11070913)
Supplement: Supplementary file 1 [file plants-11-00913-s001.zip › plants-1652499-supplementary/Table_S1.pdf]

plants MDPI

Table S1. Regenerative parameters studied in this work.

Experiment	Parameter	Time of measuremet	Units
Hydroponic	Presence/absence of callus	32, 50, 90 dap	-
Hydroponic	% of callus response	32, 50, 90 dap	%
Hydroponic	Presence/absence of AR	32, 50, 90 dap	-
Hydroponic	% of AR response	32, 50, 90 dap	%
Hydroponic	AR number	32, 50, 90 dap	-
Hydroponic	Callus stage	90 dap	-
Hydroponic	Callus area	90 dap	cm^2
Hydroponic	AR max. length	90 dap	cm
In vitro	Presence/absence of callus	7, 8, 9, 13, 15, 20, 30 dae	-
In vitro	% of callus response	7, 8, 9, 13, 15, 20, 30 dae	%
In vitro	Presence/absence of AR	7, 8, 9, 13, 15, 20, 30 dae	-
In vitro	% of AR response	7, 8, 9, 13, 15, 20, 30 dae	%
In vitro	AR number	7, 8, 9, 13, 15, 20, 30 dae	-
In vitro	AR max. length	20 dae	mm
