# Supplementary figures and images for: Effects of Auxin (Indole-3-butyric Acid) on Adventitious Root Formation in Peach-Based Prunus Rootstocks
Source: Plants (Basel). 2022 Mar 29;11(7):913. doi: 10.3390/plants11070913 (PMC9002465; doi:10.3390/plants11070913)

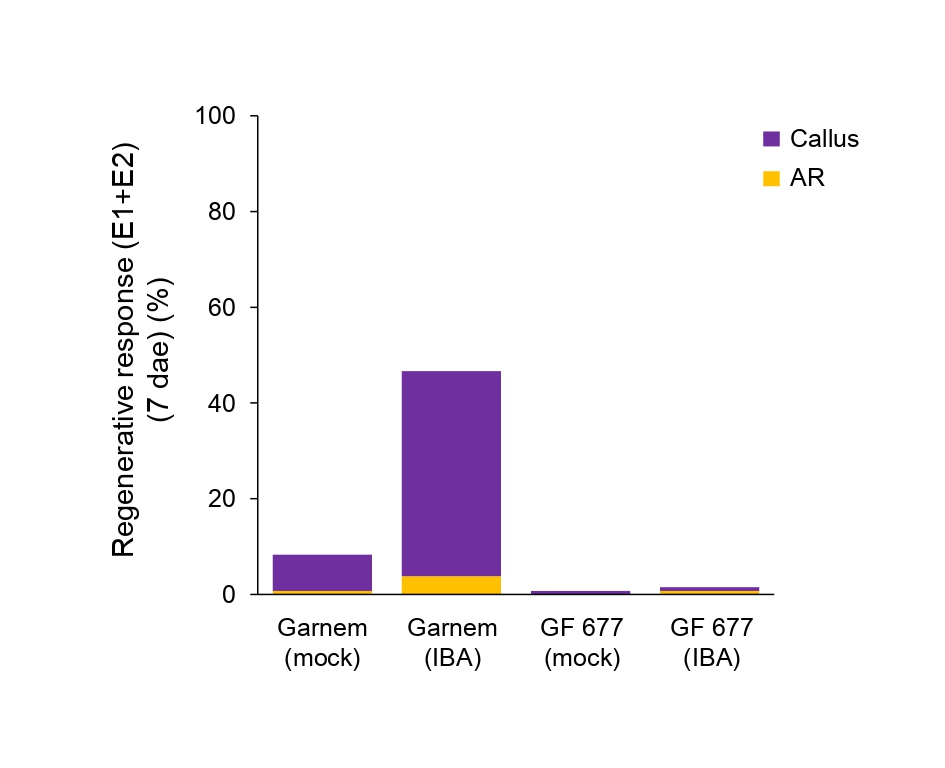

Supplement: Supplementary file 1 [file plants-11-00913-s001.zip › plants-1652499-supplementary/VideoS1_Regenerative_response.gif]

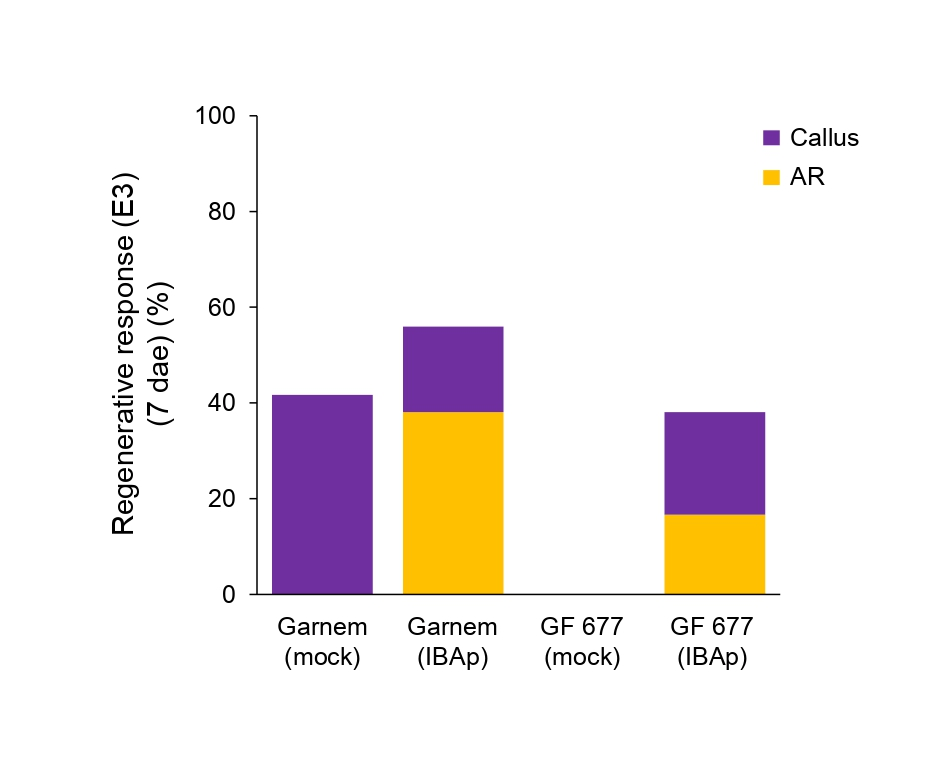

Supplement: Supplementary file 1 [file plants-11-00913-s001.zip › plants-1652499-supplementary/VideoS2_Regenerative_response.gif]
